# Portable qPCR and ddPCR Diagnostics for Cercospora Leaf Spot: Integrating Field and Laboratory Detection of *Cercospora beticola* in Beet

**DOI:** 10.3390/jof12090686

**Published:** 2026-09-12

**Authors:** Marco Crudele, Mekides Dugo Bati, Sebastiano Laera, Cataldo Laguardia, Tiziana Mascia, Francesco Faretra, Rita Milvia De Miccolis Angelini

**Affiliations:** Department of Soil, Plant and Food Sciences, University of Bari Aldo Moro, 70126 Bari, Italy; marco.crudele@uniba.it (M.C.); m.bati@phd.uniba.it (M.D.B.); sebastiano.laera@uniba.it (S.L.); az.laguardia@libero.it (C.L.); tiziana.mascia@uniba.it (T.M.); francesco.faretra@uniba.it (F.F.)

**Keywords:** pathogen detection, Swiss chard and leaf beet, sugar beet, *Beta vulgaris*, fungal disease, quantitative PCR, digital droplet PCR, field deployable assay, DNA extraction, disease management

## Abstract

*Cercospora beticola*, the causal agent of Cercospora leaf spot (CLS), is a major pathogen affecting beet (*Beta vulgaris*) worldwide. Rapid and accurate detection is critical for effective disease control. This study validated a diagnostic framework combining portable quantitative PCR (qPCR) and laboratory-based digital droplet PCR (ddPCR) assays to detect and quantify *C. beticola* in leaves, seeds, and soil-associated crop residues. We used two portable qPCR systems (Biomeme Franklin^®^ Three9 and Hyris bCUBE^™^) for in-field diagnostics, and ddPCR (Bio-Rad Laboratories) for laboratory analysis. Two rapid DNA extraction protocols, the Sigma-Aldrich REDExtract-N-Amp Plant PCR Kit (brief incubation at 95 °C) and the BN QuickPick Plant DNA Kit (magnetic-bead-based), were evaluated. The optimized portable qPCR assays achieved detection limits of 1 pg μL^−1^, PCR efficiencies of 96–102%, and R^2^ > 0.98. The ddPCR assay enabled accurate and absolute quantification even at low target concentrations and was resilient to common PCR inhibitors. Both methods proved robust in complex environmental matrices, with a detection threshold of 1% in infected residue soil. The framework’s sensitivity, specificity, and portability support timely on-site CLS diagnosis and informed management. This integrated approach advances portable molecular diagnostics and provides a platform for future field-ready disease surveillance and sustainable crop protection.

## 1. Introduction

*Cercospora beticola* Sacc. is the causal agent of Cercospora leaf spot (CLS), the most destructive foliar disease of beet (*Beta vulgaris* L.) worldwide. Its global prevalence and severe impact on Swiss chard, leaf beet, and sugar beet yield and quality make it a serious threat to crop production [1]. CLS can result in economic losses of 30–40% in susceptible cultivars under favorable conditions [2]. The pathogen is spread mainly by conidia via wind, rain, and irrigation, and it can survive in plant debris as desiccation-resistant pseudostromata for up to nine months, enabling primary infections in the subsequent season [3]. Other inoculum sources include contaminated seeds, alternative hosts, and dispersal of infected crop residues through farming practices and natural processes [4,5]. The disease develops rapidly in warm, humid environments and often co-occurs with other pathogens, such as *Phoma betae* A.B. Frank, making diagnosis and management more complex [5]. Fungicide resistance is increasing in *C. beticola* populations, further complicating control [6]. These challenges highlight the urgent need for rapid, accurate, field-ready diagnostic tools to detect the pathogen before symptoms appear. Early detection is crucial for effective and sustainable disease control.

Traditional diagnostic methods, such as visual inspection and morphological identification, are time-consuming and lack quantitative precision [4]. Molecular approaches based on polymerase chain reaction (PCR) have been developed to identify *Cercospora* spp. [7] and to specifically detect *C. beticola* using species-specific primers targeting the calmodulin gene [8]. Among molecular diagnostic techniques, loop-mediated isothermal amplification (LAMP) and recombinase polymerase amplification (RPA) offer key advantages for field applications, as they operate under isothermal conditions and can be performed with relatively simple instrumentation [9,10]. However, these methods may be more susceptible to non-specific amplification, may require additional strategies to achieve reliable quantitative information, and their performance can vary with assay design and sample matrix. In contrast, real-time quantitative PCR (qPCR) provides highly specific and sensitive detection, along with direct quantitative information through fluorescence-based monitoring of amplification. It is therefore widely considered a reference method for plant pathogen detection and quantification [10]. The advent of portable qPCR instruments has extended these benefits to field diagnostics, enabling rapid, quantitative, and potentially on-site detection of plant pathogens [11]. Compared to emerging methods such as CRISPR/Cas-based isothermal amplification, RPA, microneedle-based sampling, and smartphone-based deep learning models [12,13,14,15,16], portable qPCR platforms uniquely combine field portability with the proven analytical performance and quantitative capabilities of conventional qPCR. These characteristics provide a strong rationale for evaluating portable qPCR platforms for rapid, reliable, and quantitative pathogen detection in field settings.

Despite technological advances, there remains a scarcity of highly validated, sensitive, and fully portable molecular assays for major fungal pathogens, especially quantitative tools for *C. beticola*. Developing and deploying such assays is further complicated by the high genetic and genotypic diversity found within *C. beticola* populations. Both clonal reproduction and genetic recombination underlie this diversity, further amplified by substantial gene flow among geographically distant populations [17]. Genome-wide single nucleotide polymorphism (SNP) analyses have revealed shared genotypes across distinct regions, highlighting the pathogen’s capacity for long-distance dispersal [18]. This pronounced genetic variability poses a major challenge for molecular diagnostics, as sequence polymorphisms in assay target regions can compromise primer and probe binding, potentially resulting in false negatives or reduced assay sensitivity.

The development of portable qPCR devices and miniaturized fluorescence detection systems has facilitated molecular analyses outside traditional laboratory settings [19]. Nevertheless, successful field application requires efficient DNA extraction from diverse and complex matrices without compromising analytical sensitivity. Digital droplet PCR (ddPCR) enables absolute quantification of nucleic acids [20]. In ddPCR, the reaction mixture is partitioned into thousands of water-in-oil droplets, each serving as a discrete PCR microreactor [21]. After amplification, droplets are scored as positive or negative based on endpoint fluorescence, and the absolute copy number is determined using Poisson statistics [20]. ddPCR provides high precision for detecting low-abundance targets and is more tolerant of PCR inhibitors, which is particularly advantageous for pathogen detection in complex environmental samples [22,23].

This study addresses current gaps in *C. beticola* diagnostics by developing and validating portable real-time qPCR assays for rapid, in-field detection of the pathogen across diverse environmental matrices, including seeds, plant residues, and leaves. These streamlined protocols remove the need for specialized laboratory equipment. The portable assays can be used by non-experts in the field, enabling quick and reliable diagnosis to support disease management. Alongside, we establish a sensitive ddPCR protocol for laboratory use, suited to epidemiological studies and precise pathogen quantification. By integrating rapid field diagnostics with advanced laboratory quantification, our approach offers a comprehensive solution for CLS management and lays the groundwork for broader use of portable molecular tools in plant pathology.

## 2. Materials and Methods

### 2.1. Fungal Strains and Growing Conditions

*C. beticola* isolates were obtained from *Beta vulgaris* subsp. *vulgaris* leaves collected at multiple field sites in the Apulia region, Southern Italy. Molecular identification was performed by PCR with species-specific primers [8], and species identity was confirmed via Sanger sequencing (Macrogen Europe B.V., Amsterdam, The Netherlands; Milan Genome Centre, Milan, Italy). Specificity was assessed against a panel of 13 fungal and bacterial isolates: *Rhizopus* sp., *Aspergillus niger* Tiegh., *Colletotrichum* sp., *Botrytis cinerea* Pers., *Alternaria alternata* (Fr.) Keissl., *Penicillium digitatum* (Pers.) Sacc., *Cladosporium* sp., *Macrophomina phaseolina* (Tassi) Goid., *Trichoderma asperellum* Samuels, Lieckf. and Nirenberg, *Fusarium* spp., *P. betae*, and bacterial isolates of *Pseudomonas syringae* (van Hall) Migula and *Xanthomonas* spp. All isolates were stored in 10% glycerol at −80 °C and subcultured on potato dextrose agar (PDA; prepared by infusing 200 g of peeled potatoes incubated for 1 h at 60 °C, 20 g dextrose (Carlo Erba reagents S.r.l., Cornaredo, Italy), pH 6.5, and 20 g agar Oxoid No. 3 per litre) or nutrient agar (NA; 8 g nutrient broth and 20 g agar Oxoid No. 3 per litre) for maintenance and preparation of fresh cultures.

### 2.2. Plant Material

Swiss chard and leaf beet samples and soil-associated crop residues were systematically collected from several field sites to represent a range of growing conditions and infection pressures. This strategy allowed for comprehensive assessment of PCR assay sensitivity and specificity across diverse sample types. Materials collected included naturally infected leaves (30–80% symptomatic leaf area), healthy control leaves from pathogen-free locations, and soils from fields with a known history of CLS. Additionally, to evaluate the rapid DNA extraction protocol for seed matrices and seedborne inoculum detection, we analysed 13 B. vulgaris seed lots (both commercial and farmer-saved, collected in 2024 and 2025; Table S3).

### 2.3. DNA Extraction Methods

To enable both laboratory and in-field detection, multiple DNA extraction methods were systematically assessed using pure cultures, seeds, fresh leaves, and crop residues with soil. Genomic DNA (gDNA) was extracted from fungal isolates cultured for three days on PDA and from 2 mL NA bacterial cultures, following standard protocols [24,25]. DNA was also extracted from a pure culture of *C. beticola*, which served as a positive control in assay validations, and from healthy leaves, which served as negative controls (NC). All gDNA samples, including those used for standard curve preparation, were treated with DNase-free pancreatic RNase (Sigma-Aldrich, Milan, Italy) according to the manufacturer’s instructions to remove RNA contamination.

**Figure 1:**
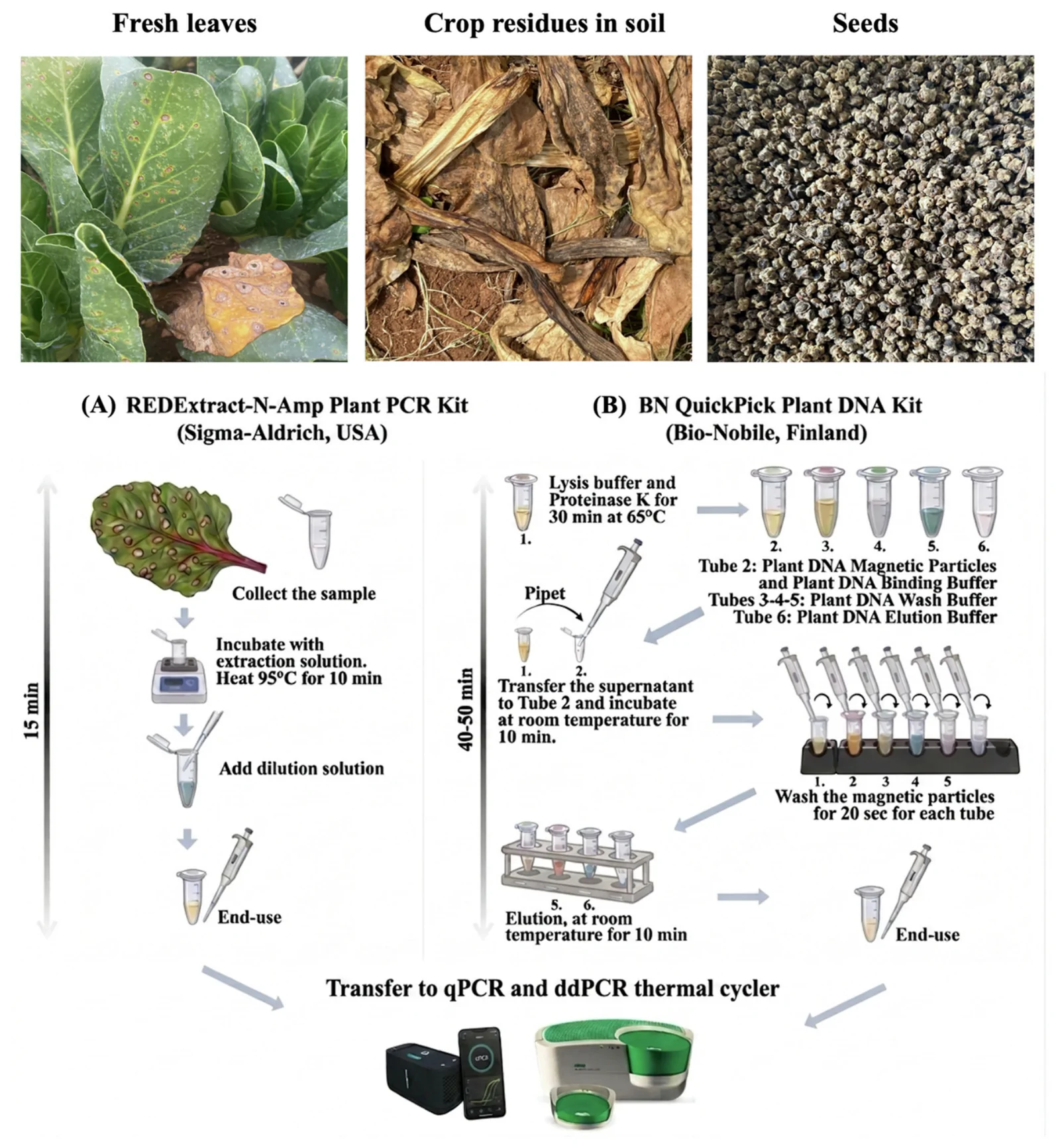
Comparative overview of rapid DNA extraction workflows across plant matrices, showing main steps and processing times for two protocols: (**A**) heat-based lysis and dilution and (**B**) magnetic bead-based enzymatic lysis. Vertical arrows indicate total hands-on time from homogenization to analysis.

For field applications, two rapid DNA extraction protocols were tested in parallel with the standard method: the REDExtract-N-Amp Plant PCR Kit (Sigma-Aldrich, St. Louis, MO, USA; protocol A) and the BN QuickPick Plant DNA Kit (Bio-Nobile, Turku, Finland; protocol B) (Figure 1). To ensure consistent tissue disruption and maximize DNA yield, seeds were ground using a battery-powered portable drill (Dremel Lite 7760, Robert Bosch Tool Coorporation, Mount Prospect, IL, USA).

For protocol A, 8 mg of sample was incubated in 100 µL extraction solution, vortexed, heated at 95 °C for 10 min, and then mixed with 100 µL dilution solution for direct PCR. For seeds, all volumes were doubled to standardize reagent-to-tissue ratios. Protocol B involved lysing 4 mg of sample in 75 µL lysis buffer with 5 µL Proteinase K, vortexing, and incubating at 65 °C for 30 min. Lysates were centrifuged (5 min, 18,000 rpm), and the supernatant was transferred to a 96-well plate. Magnetic beads (5 µL) and binding buffer (125 µL) were added, mixed by pipetting, and incubated on a magnetic plate for 5–10 min at room temperature. Beads were washed three times with 250 μL wash buffer, each wash followed by mixing and a 10-min incubation. DNA was eluted in 50 µL elution buffer, mixed, incubated for 10 min on the magnetic plate, and collected for analysis. The specific sample input amounts for protocols A and B were established based on preliminary optimization trials. These values were selected to achieve an optimal balance between maximizing target DNA yield and minimizing the co-extraction of PCR inhibitors from complex sample matrices, aligning with best practices for environmental sample processing [26,27].

DNA extracts were quantified using a NanoDrop 2000 spectrophotometer (Thermo Fisher Scientific Inc., Wilmington, DE, USA) and a Qubit 2.0 fluorometer with the Qubit dsDNA High Sensitivity Assay Kit (Life Technologies, Carlsbad, CA, USA). Extracted DNA was then used as a template in qPCR and ddPCR assays to assess the compatibility, sensitivity, and reproducibility of each extraction protocol.

To ensure extraction reliability and monitor for potential PCR inhibitors, especially in seeds and soil-containing crop residues, standardised positive controls were included. Fungal genomic DNA (gDNA) was spiked into extracts from crop residues, soil, and seed samples as an internal reference. All extractions were performed in triplicate.

### 2.4. Setup and Optimization of qPCR and ddPCR Assays

gDNA from *C. beticola* isolates was used to optimize both qPCR and ddPCR assays. For both assays, the same primer and TaqMan probe set targeting the calmodulin gene of *C. beticola* was used, as described by Knight et al. [8] (Table S1). The TaqMan probe was labelled at the 5′ end with a FAM fluorophore, and both primers and probe were synthesized and HPLC-purified by Macrogen (Seoul, Republic of Korea). In silico analysis using NCBI Primer-BLAST [28] confirmed no cross-reactivity with non-target genomic sequences or off-target organisms. To determine optimal annealing temperatures, thermal gradients of 60, 62, and 64 °C were tested using 10 ng·µL^−1^ gDNA from pure *C. beticola* cultures. Assay performance was assessed by Cq values in qPCR and fluorescence amplitude separation in ddPCR, selecting conditions minimizing intermediate fluorescence droplets. Experimental design, procedures, and reporting followed the MIQE [29] and dMIQE [30,31] guidelines for reproducibility and transparency. All experiments were conducted in triplicate, with a no-template control (NTC; ultrapure sterile water) included in every run.

#### 2.4.1. qPCR Assays

qPCR assays were conducted using two portable platforms: the Franklin^®^ Three9 thermocycler (Biomeme, Inc., Philadelphia, PA, USA; referred to as platform 1) and the Hyris bCUBE™ system (Hyris Ltd., London, UK; platform 2). Reaction mixtures (20 μL final volume) consisted of 1× master mix (LyoDNA™ 2.0 + IPC Master Mix, Biomeme, Inc. for platform 1; Sso Advanced™ Universal Probes Supermix, Bio-Rad Laboratories, Hercules, CA, USA, for platform 2), 250 nM each primer, 150 nM probe, 1 μL DNA template, and ultrapure water. For platform 1, thermal cycling involved an initial denaturation at 95 °C for 1 min, followed by 45 cycles of 95 °C for 1 s and annealing/extension at 60–64 °C for 20 s. For platform 2, the protocol was 95 °C for 3 min, then 40 cycles of 95 °C for 10 s and 60–64 °C for 30 s. All reagents and protocols were used according to manufacturers’ instructions to ensure reproducibility (Biomeme, Inc., 2025; Bio-Rad Laboratories, 2025).

#### 2.4.2. ddPCR Assay

ddPCR was performed on the QX200 Droplet Digital PCR system (Bio-Rad Laboratories). Each 22 µL reaction mixture contained 1× ddPCR™ Supermix for probes (No dUTP), primers (500 nM), probe (250 nM), 1 µL DNA template, and nuclease-free water, combined with 70 µL droplet generation oil in an 8-channel cartridge. Droplet generation was carried out using the QX200 Droplet Generator. Subsequently, 40 µL of the resulting emulsion was transferred to ddPCR 96-Well PCR Plates, sealed with pierceable foil, and amplified in a T100™ Thermal Cycler. The cycling protocol consisted of an initial denaturation at 95 °C for 10 min, followed by 40 cycles of 94 °C for 30 s and 60–64 °C for 1 min (2 °C/s ramp rate), and a final signal stabilization step at 98 °C for 10 min. Following amplification, emulsified samples were analysed with a QX200 droplet reader to quantify fluorescence signals from individual droplets. All procedures strictly followed manufacturer guidelines to ensure reproducibility and minimize technical bias (Bio-Rad Laboratories, 2025).

### 2.5. Validation of the Assays

Analytical sensitivity and specificity were systematically evaluated for all qPCR and ddPCR assays to ensure robust detection of *C. beticola* in both field and laboratory conditions across diverse environmental and plant materials, following established recommendations for molecular diagnostics [29,32]. Specificity was determined using 1 ng of DNA from twenty representative *C. beticola* isolates collected from seven distinct fields in the Apulia region, accounting for potential intraspecific genetic variation, and from an extensive panel of non-target fungal and bacterial species. Healthy beet DNA served as a negative control. Analytical sensitivity was assessed by testing ten-fold serial dilutions (from 1 fg·µL^−1^ to 1 ng·µL^−1^) of *C. beticola* gDNA.

To evaluate potential PCR inhibition caused by co-extracted matrix compounds, inhibition tests were performed on both qPCR (platform 1) and ddPCR platforms using soil and seed row extracts obtained via protocol A, as recommended for environmental DNA studies [26,27]. Purified *C. beticola* gDNA was serially diluted ten-fold (from 1 ng μL^−1^ to 0.1 fg μL^−1^), and 1 μL of each dilution was spiked into 1 μL of raw DNA extract from pathogen-free soil and seed matrices. All matrices were previously confirmed as negative for *C. beticola* using both qPCR and ddPCR. Serial dilutions of pure fungal DNA mixed with molecular-grade water served as baseline controls.

To further assess the analytical sensitivity of qPCR assays in complex environmental matrices and evaluate the impact of PCR inhibitors, we applied the methods to soil samples artificially spiked with varying amounts of infected plant residues, as described in related environmental DNA detection protocols [33]. Pathogen-free soil was used as the base matrix. The inoculum consisted of *C. beticola*-infected plant material exhibiting 75–80% symptomatic leaf area. Infected tissue was lyophilized with a Lio 5P freeze-dryer (Cinque Pascal, Trezzano sul Naviglio, Italy) for 48–72 h to ensure complete dehydration and standardization. Detection limits were established by preparing a dilution series: lyophilized, finely ground infected tissue was blended with pathogen-free soil to a total weight of 1 g, covering contamination levels of 100%, 75%, 50%, 25%, 10%, 5%, 1%, 0.5%, 0.1%, and 0.05% (down to 0.5 mg infected material in 999.5 mg soil). These samples were used to validate DNA extraction efficiency and assay detection limits in soil. Each dilution was tested in triplicate to ensure statistical robustness. PCR inhibition was quantified by calculating the Cq shift (ΔCq) between spiked matrix samples and corresponding pure fungal DNA controls.

Finally, method validation was performed on seed and soil samples of diverse origin, naturally infected or contaminated with *C. beticola*, in accordance with best practices for diagnostic assay validation [32]. Method accuracy was evaluated for repeatability by calculating the intra-assay coefficient of variation (CV).

### 2.6. Data Processing and Statistical Analysis

Data analysis for qPCR platform 1 was performed using the Biomeme Go application (version 8.0.1, Biomeme, Inc., Philadelphia, PA, USA), and results from platform 2 were processed with the Hyris bAPP web-based software (version 3.2.0, Hyris Ltd., London, UK). Both platforms utilized automated thresholds to determine quantification cycle (Cq) values consistently across all samples, minimizing operator bias (Biomeme, Inc., 2025; Hyris Ltd., 2025). Absolute copy number concentrations for ddPCR were calculated using Poisson statistics with 95% confidence intervals in QuantaSoft software (version 1.7.4) and QX Manager Standard Edition (Bio-Rad Laboratories, 2025). Only samples with more than 12,000 events were considered valid. For each experiment, fluorescence amplitude thresholds were manually set using No Template Control (NTC) baselines to distinguish positive from negative droplets, ensuring methodological consistency and scientific rigor (Bio-Rad Laboratories, 2025).

Standard curves were generated by plotting the logarithm (Log_10_) of DNA concentration against Cq values for qPCR or absolute copy number for ddPCR, in accordance with established protocols [29,30]. The coefficient of determination (R^2^) and the slope of each standard curve were calculated to assess amplification efficiency (E), defined as E = [10^(−1/slope)^ − 1] × 100. Statistical analyses were conducted using OriginPro, Version 2025b (OriginLab Corporation, Northampton, MA, USA), and R version 4.5.1 (R Foundation for Statistical Computing, Vienna, Austria, 2025) within RStudio version 2025.05.1+513 (Posit Software, PBC, Boston, MA, USA). Graphical outputs were generated with the ggplot2 package, and linear models were fitted using the lm() function to maintain reproducibility and transparency in data processing.

Comparative analysis between portable qPCR platforms and ddPCR reference data was conducted using Bland–Altman analysis to assess systematic bias and determine 95% Limits of Agreement (LoA) [34]. For direct comparison, Cq values from qPCR were converted to log_10_ copy numbers using regression equations derived from ddPCR standard curves. The probabilistic Limit of Detection (LOD) was estimated by probit regression analysis, identifying the DNA concentration corresponding to a 95% detection probability [35]. One-way analysis of variance (ANOVA), followed by Tukey’s Honestly Significant Difference (HSD) test, was performed to evaluate significant differences (*p* < 0.05) among annealing temperatures and DNA extraction methods. This integrated approach reinforced the statistical rigor and ensured comparability of results across platforms.

## 3. Results

### 3.1. Optimization of qPCR and ddPCR Assays

The qPCR and ddPCR assays were optimized to determine the optimal annealing temperature for *C. beticola* detection. All tested temperatures supported successful amplification of *C. beticola* template DNA, with no fluorescent signal in NCs or NTCs. In qPCR, one-way ANOVA revealed a significant effect of temperature on Cq values (*p* < 0.001). Tukey’s HSD post hoc analysis identified 62 °C as the optimal condition, yielding significantly lower mean Cq values and higher fluorescence intensity than 60 °C or 64 °C (Table 1; Figure S1A). The low standard deviations among technical triplicates confirmed the protocol’s robustness and reproducibility. Conversely, ddPCR absolute quantification remained statistically unchanged across the 60–64 °C range (*p* = 0.570, Table 1). However, visual inspection of 1D amplitude plots showed that 62 °C provided the best separation between positive and negative droplet populations, minimizing rain droplets that could compromise threshold accuracy (Figure S1B). Thus, while ddPCR is resilient to small temperature variations, 62 °C maximizes the signal-to-noise ratio for both platforms. Accordingly, 62 °C was adopted as the standard annealing temperature for all subsequent qPCR and ddPCR experiments.

### 3.2. Limits of Detection and Specificity of the Assays

**Figure 2:**
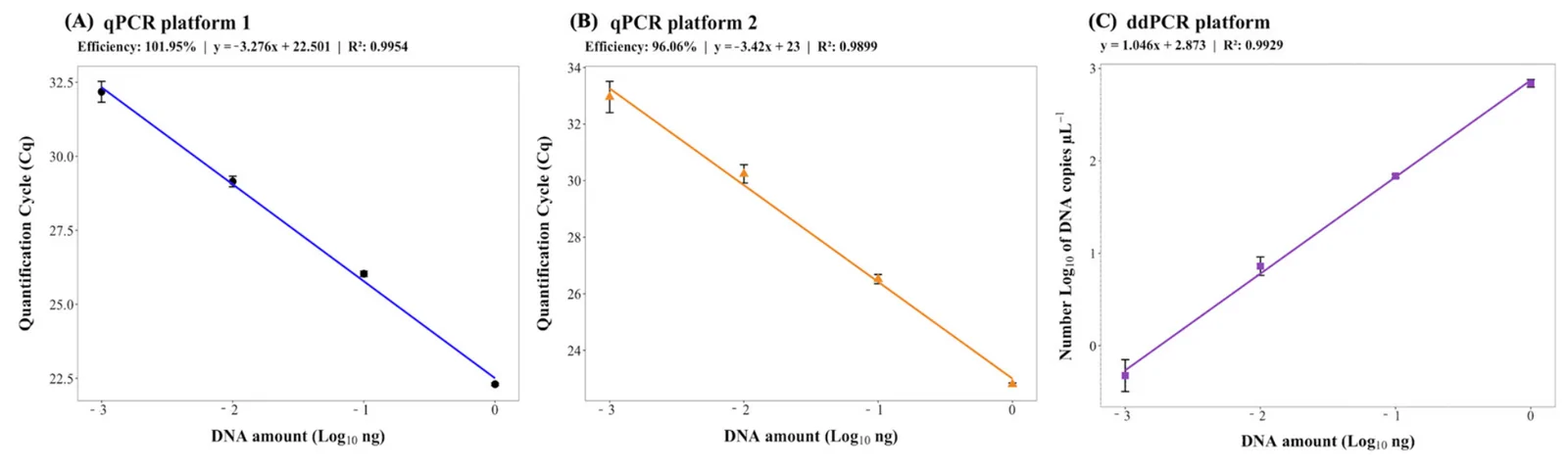
Standard curves generated from ten-fold serial dilutions of *C. beticola* gDNA (1 ng µL^−1^ to 1 fg µL^−1^) for qPCR and ddPCR assays. (**A**) qPCR platform 1: Cq vs. DNA concentration; (**B**) qPCR platform 2: Cq vs. DNA concentration; (**C**) ddPCR: measured vs. theoretical copy number. Data represent means of three technical replicates; error bars indicate standard deviation.

Analytical sensitivity was assessed using ten-fold serial dilutions of *C. beticola* gDNA ranging from 1 ng μL^−1^ to 1 fg μL^−1^. On qPCR platform 1, the assay showed a strong linear correlation between Cq values and DNA concentration from 1 ng μL^−1^ to 1 pg μL^−1^ (R^2^ = 0.998; slope = −3.276; PCR efficiency = 101.95%; Figure 2A). The limit of detection (LOD) for reliable quantification was 1 pg μL^−1^. For qPCR platform 2, a similar LOD of 1 pg μL^−1^ was observed (mean Cq = 33.26 ± 0.73), with results highly consistent with platform 1 (R^2^ = 0.9982; slope = −3.42; PCR efficiency = 96.06%; Figure 2B). The ddPCR assay also enabled reliable quantification down to 1 pg μL^−1^ (approximately 0.50 copies μL^−1^). Linear regression analysis confirmed a strong correlation (R^2^ = 0.9929) between log_10_ theoretical DNA concentrations and Poisson-measured copies (slope = 1.046), demonstrating quantitative accuracy (Figure 2C).

**Figure 3:**
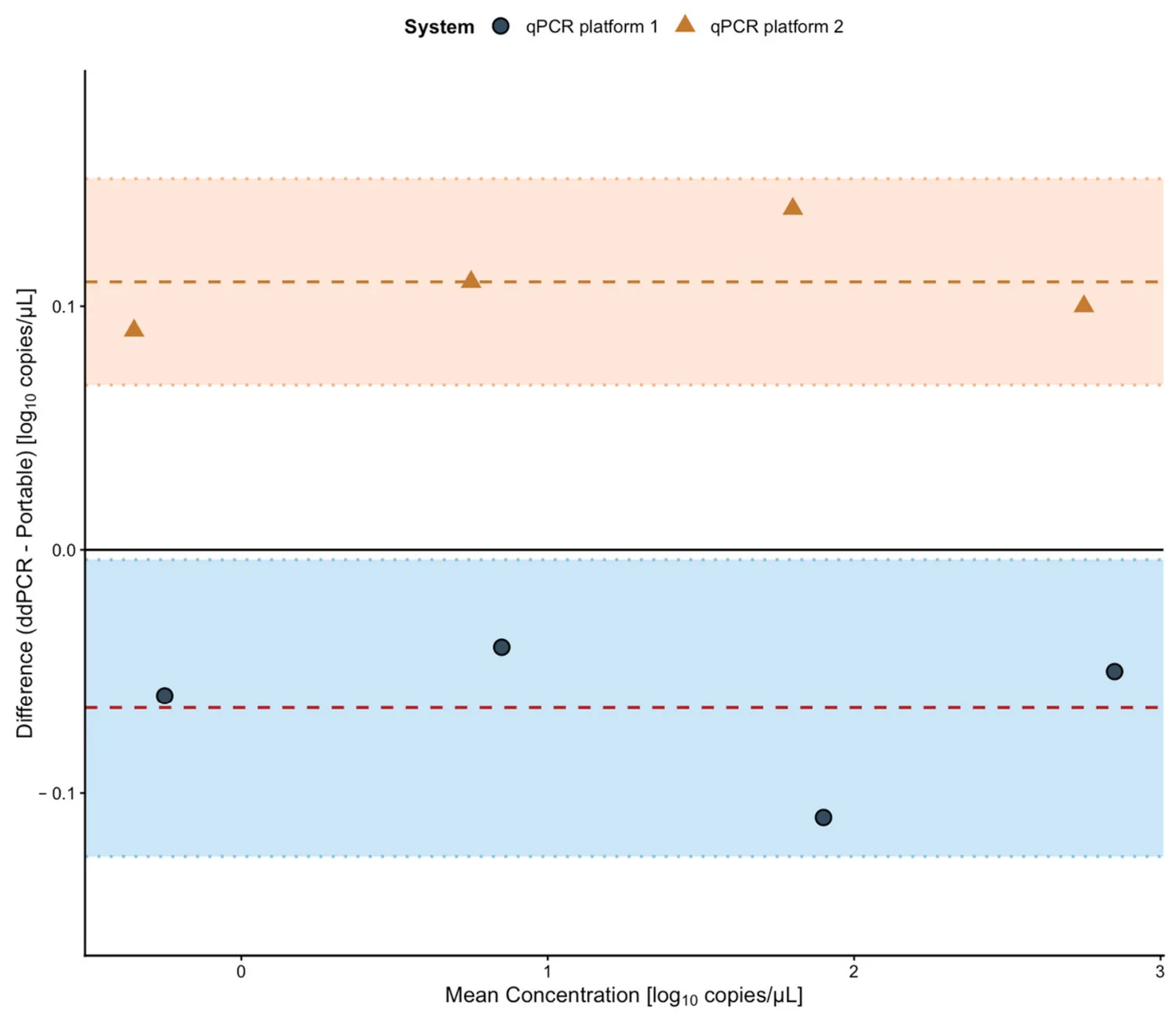
Bland–Altman plot comparing agreement between the portable qPCR platforms (qPCR platform 1, dark blue circles; qPCR platform 2, orange triangles) and the laboratory-based ddPCR method. The x-axis shows the mean log_10_ copy number (copies µL^−1^) calculated for the two methods, and the y-axis displays the difference in log_10_ copy number between ddPCR and portable qPCR. Dashed lines represent the mean bias for each system, while shaded areas indicate the 95% Limits of Agreement (LoA).

Cq values were converted to absolute concentrations using standard curve interpolation and analysed with Bland–Altman plots, revealing high concordance between both portable qPCR platforms and the ddPCR reference (Figure 3). The mean biases for qPCR platform 1 (−0.065 log10 copies μL^−1^) and platform 2 (+0.110 log10 copies μL^−1^) were negligible, with all data points residing within the 95% limits of agreement, in line with the stringent requirements for quantitative assay validation. These results reinforce the reliability of portable qPCR platforms for precise DNA quantification.

**Figure 4:**
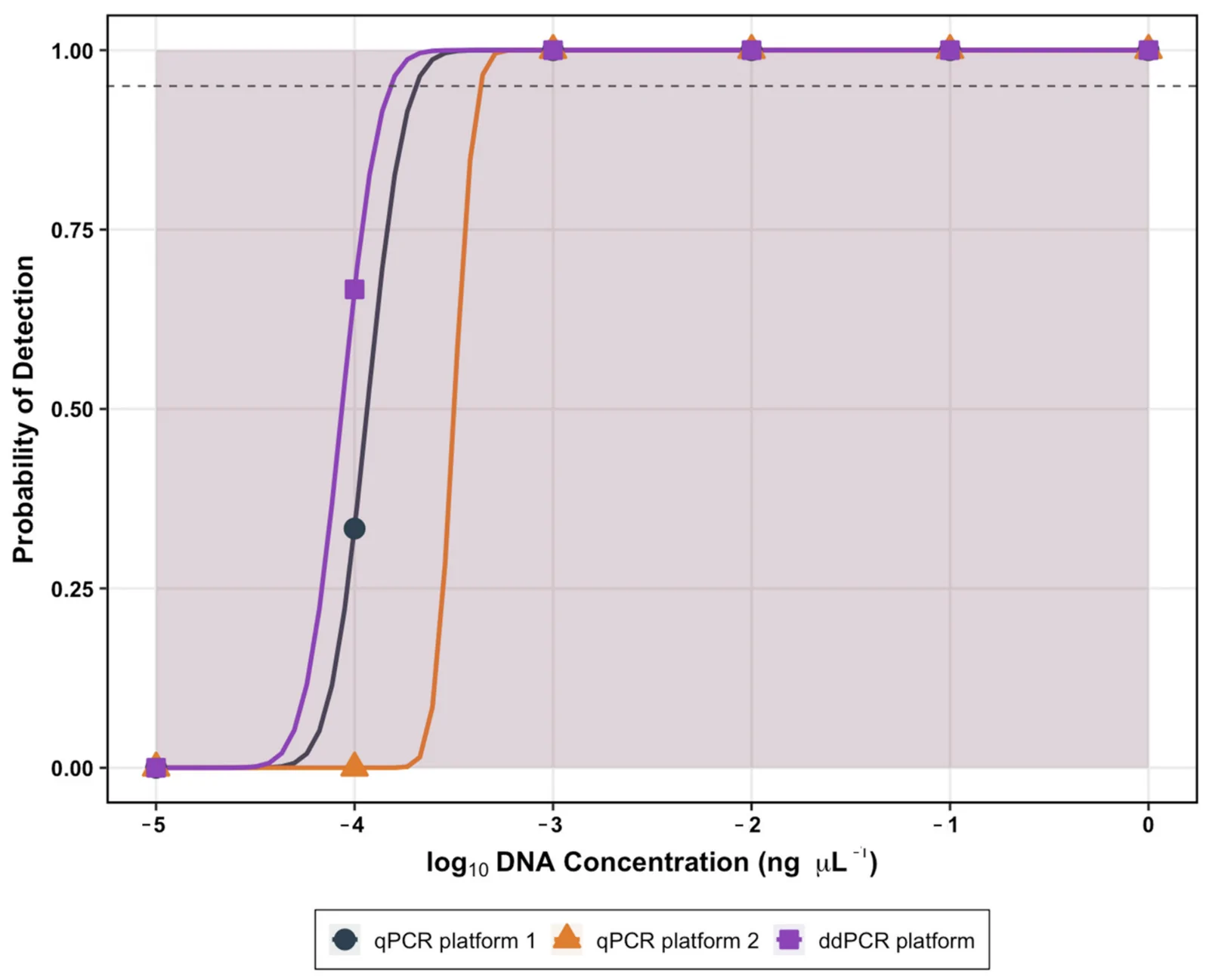
Probit regression models showing the 95% detection probability (limit of detection, LOD) for qPCR platform 1 (grey circles), qPCR platform 2 (orange triangles), and ddPCR platform (purple squares). The x-axis represents the log10-transformed DNA concentration (ng µL^−1^), and the y-axis indicates the probability of detection. The dashed horizontal line denotes the 95% threshold used to determine LOD. The LOD was 1 pg μL^−1^ for each platform, as determined by the 95% detection probability.

Furthermore, probit regression analysis demonstrated that all platforms achieved a 95% probability of detection at the established LOD, consistent with previous findings on molecular assay performance. Importantly, ddPCR provided superior accuracy and reproducibility at the lowest DNA concentrations tested (Figure 4).

The specificity of the primer and probe sets was rigorously evaluated against a panel of 13 phylogenetically diverse fungal and bacterial species. No amplification was detected in either portable qPCR platform when testing non-target organisms, and ddPCR measurements for these species remained below 0.2 copies μL^−1^, consistent with background noise and confirming the high specificity of the assays. These results fulfil established criteria for molecular diagnostics, minimizing the risk of false positives and ensuring reliable pathogen detection. In positive control reactions using target DNA, both qPCR platforms produced consistent Cq values, while ddPCR provided reliable absolute quantification, highlighting the reproducibility and quantitative robustness of each platform. Additionally, field validation using qPCR verified accurate detection of all 20 representative *C. beticola* isolates from seven Apulian fields (Cq range: 17.89–19.34 with platform 1), underscoring the assay’s diagnostic reliability, sensitivity, and field applicability (Figure S2).

### 3.3. Comparative Assessment of Field-Deployable DNA Extraction Protocols

**Figure 5:**
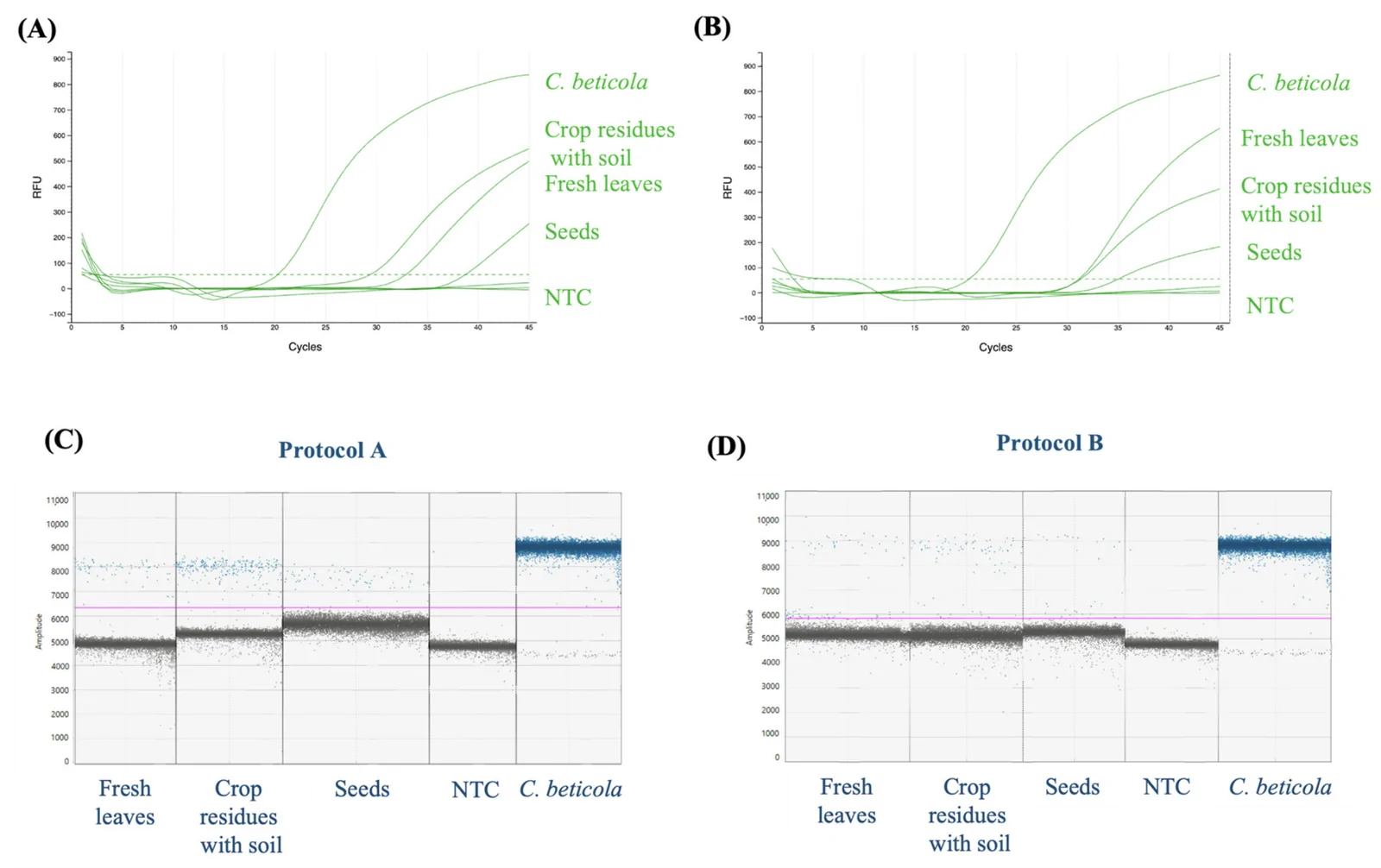
Comparative analysis of rapid DNA extraction methods using real-time PCR (qPCR) and digital droplet PCR (ddPCR) across multiple sample types. Genomic DNA from a pure *C. beticola* colony extracted using the standard CTAB protocol served as a positive control. (**A**,**B**) Lines represent qPCR amplification curves for DNA extracted with (**A**) the REDExtract-N-Amp Plant PCR Kit (protocol A, Sigma-Aldrich) and (**B**) BN QuickPick Plant DNA Kit (protocol B, Bio-Nobile). (**C**,**D**) ddPCR 1D amplitude plots for DNA extracted using (**C**) protocol A and (**D**) protocol B. In ddPCR, the horizontal pink line denotes the fluorescence amplitude threshold distinguishing positive droplets (blue) from negative droplets (grey). No fluorescence signals or positive partitions were detected in the non-template controls (NTC).

Two rapid DNA extraction protocols were evaluated across three sample matrices: fresh leaves, crop residues with soil, and seeds. DNA yields, measured by fluorometric quantification, were comparable for both protocols, with protocol B producing slightly higher dsDNA concentrations. Protocol B demonstrated significantly higher 260/280 ratios, indicating more effective protein removal, while 260/230 ratios varied by sample matrix, reflecting differences in co-purified contaminants (Figure 5; Table S2). Despite these differences in extract purity, downstream qPCR amplification using portable platform 1 remained consistent, as shown by stable Cq values for both protocols and the absence of PCR inhibition, confirmed by internal positive controls. Diagnostic accuracy was comparable between protocols, with observed Cq shifts resulting from variations in template DNA quantity rather than extract quality. Absolute quantification by ddPCR supported these findings. The observed differences in positive droplet fluorescence amplitudes between protocol A and protocol B (Figure 5C,D) are primarily due to variations in extract composition. Specifically, the less refined extraction process in protocol A leaves residual matrix compounds, which can slightly increase baseline fluorescence. Nevertheless, this effect does not affect the binary classification of partitions or compromise quantification accuracy [22]. Notably, protocol A consistently yielded higher *C. beticola* target copy numbers than protocol B across all matrices, indicating greater enrichment of amplifiable target DNA, especially in complex matrices such as soil. Late-cycle qPCR signals exceeding the diagnostic threshold were classified as negative, consistent with accepted detection limits. Overall, although protocols A and B differ in purity profile and matrix-specific amplification kinetics, their diagnostic performance is comparable. Protocol B provides advantages in protein removal and reproducibility, facilitating laboratory automation, whereas protocol A offers robust performance with a streamlined, field-deployable workflow.

### 3.4. Validation and Detection Limits in Artificially Inoculated Soil and Seed Samples

**Figure 6:**
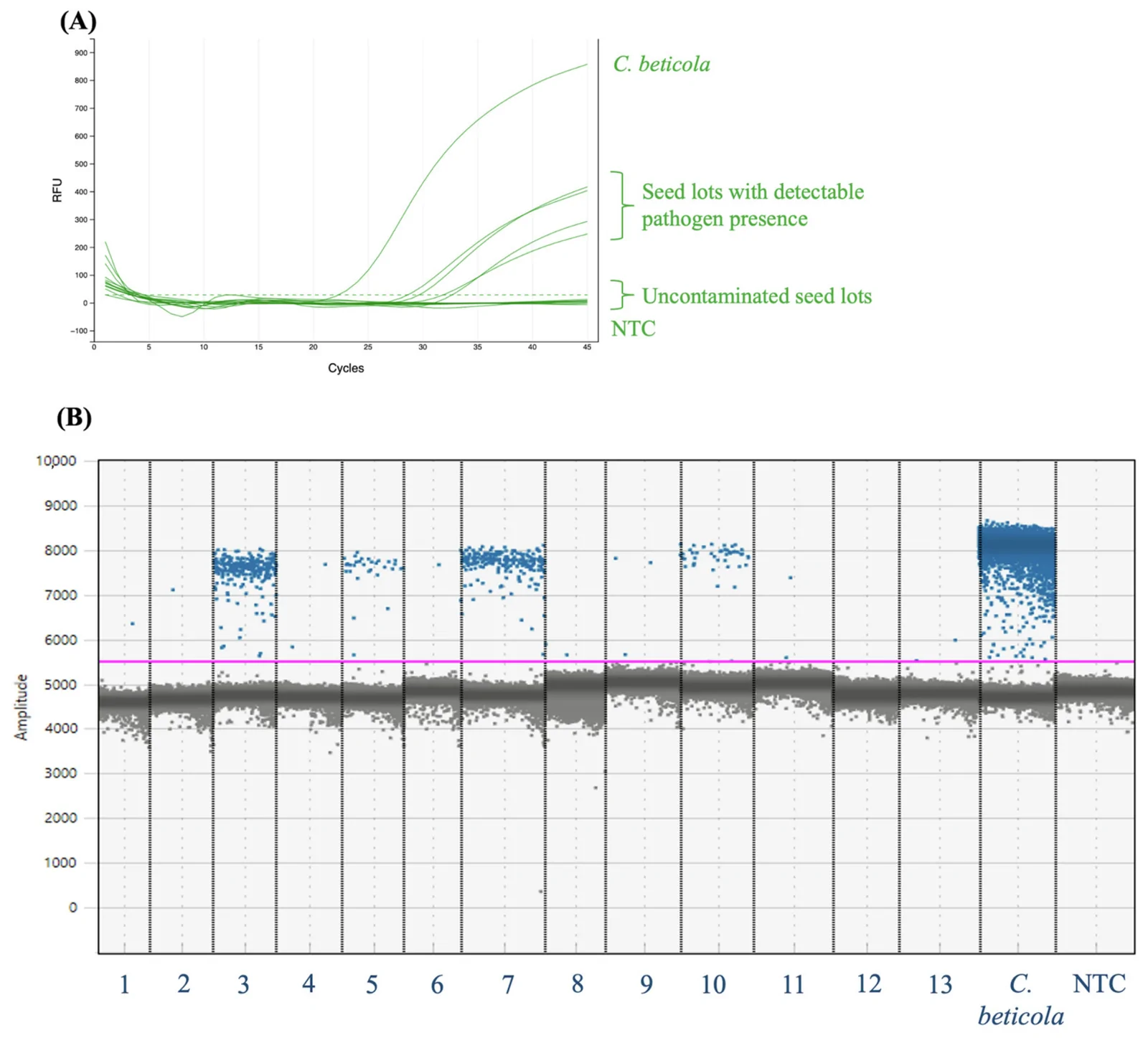
qPCR (**A**) and ddPCR (**B**) detection of *C. beticola* in commercial and farmer-saved leaf beet seed lots after rapid DNA extraction (protocol A). Genomic DNA from a pure *C. beticola* colony (CTAB extraction) served as a positive control. In panel A, lines represent qPCR amplification curves for each sample category (as labeled). In ddPCR amplitude plots, the pink threshold line separates positive (blue) from negative (grey) droplets. No signals were detected in non-template controls.

Matrix-induced PCR inhibition was assessed in two independent assays, both demonstrating high assay robustness. Amplification profiles and Cq values for pure *C. beticola* DNA were consistent with those obtained from DNA spiked into crude soil and seed extracts, indicating minimal matrix interference. In the soil interference assay, pure DNA yielded mean Cq values of 23.10 ± 0.08 (1 ng μL^−1^) and 30.40 ± 0.31 (0.01 ng μL^−1^); after spiking into crude soil extracts, values remained stable at 23.44 ± 0.13 and 30.86 ± 0.28, respectively. In the seed inhibition assay, pure DNA produced mean Cq values of 23.08 ± 0.28 (1 ng μL^−1^) and 31.98 ± 0.82 (0.01 ng μL^−1^), while spiking into crude seed extracts yielded similar values of 23.30 ± 0.12 and 31.64 ± 0.49. For both matrices, extreme dilutions led to late amplification (Cq > 36) or no amplification (Cq = 0), further substantiating the established practical limit of detection (pLoD). Across all reliable data points, the maximum observed shift in quantification cycles (ΔCq) was minimal (≤0.46 cycles), supporting the assay’s precision and reproducibility. Screening of 13 seed lots using protocol A showed high reproducibility in DNA yield and detection sensitivity, validated by both qPCR and ddPCR, which detected *C. beticola* contamination in the two farmer-saved samples and in two of the commercial ones (Figure 6; Table S3). Conversely, when evaluating environmental soil samples of different origins for natural contamination, all analysed samples were negative, with target DNA concentrations remaining below the established limits of analytical sensitivity.

**Figure 7:**
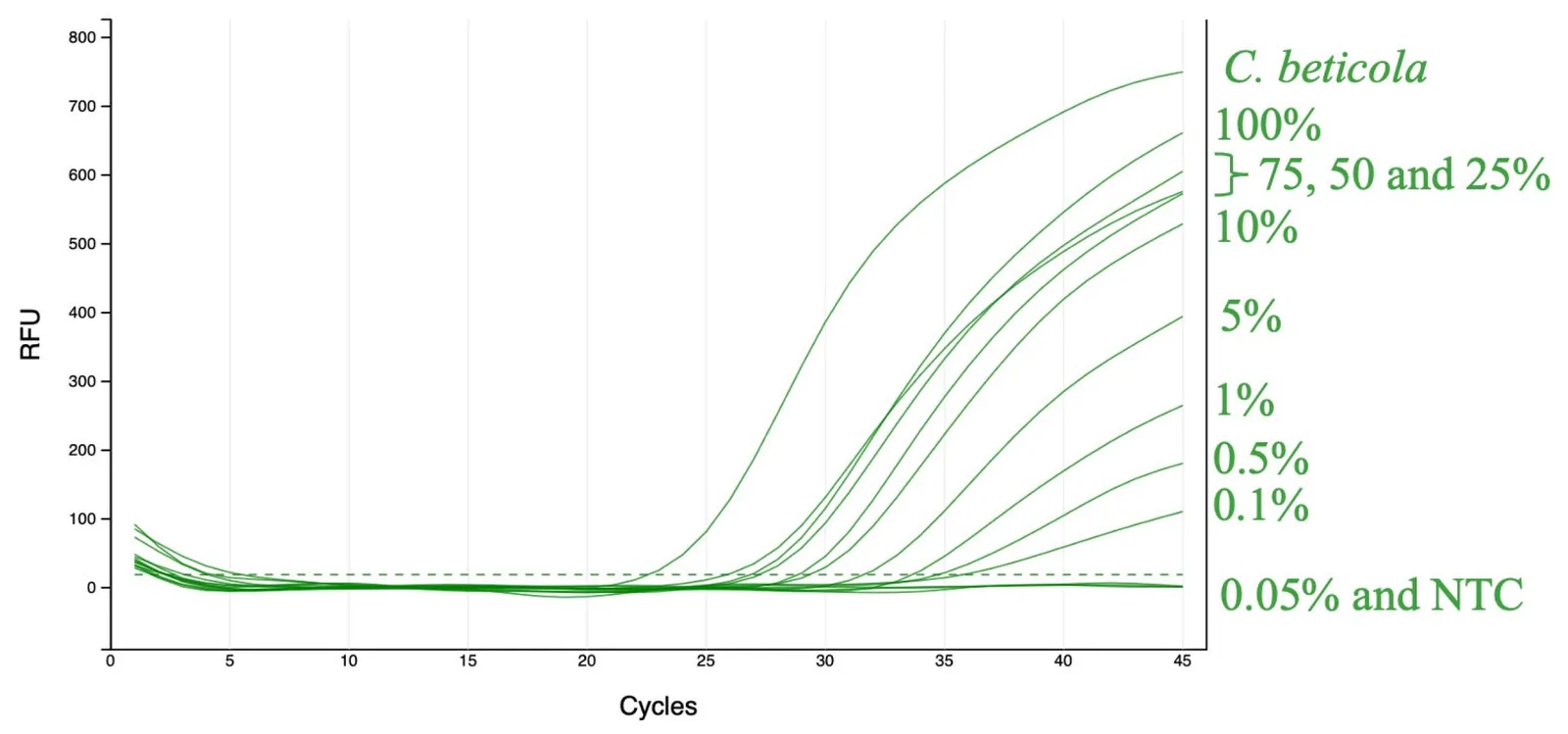
Detection thresholds for *C. beticola* in artificially inoculated soil. Lyophilized infected leaves were mixed into pathogen-free soil to generate contamination levels from 100% to 0.05%. Lines represent qPCR amplification curves for each contamination level (as labeled). DNA was extracted using protocol A and analysed on qPCR platform 1. Genomic DNA from a pure *C. beticola* colony (CTAB extraction) served as a positive control (Cq 22.55). No fluorescence was detected in non-template controls (NTC). Cq values were calculated using a fixed fluorescence threshold of 18.91 RFU on a Biomeme Franklin^®^ Three9 thermocycler.

To evaluate detection capabilities in complex soil matrices and establish detection thresholds, we conducted a dilution series experiment by mixing *C. beticola*-infected plant material with pathogen-free soil at concentrations from 100% down to 0.05%. Amplification profiles (Figure 7) and corresponding Cq values (Table S4) from samples analysed using optimized protocol A on qPCR platform 1 displayed the expected logarithmic response, confirming assay sensitivity. The progressive delay in amplification with decreasing target DNA concentrations indicated that template availability was the primary limiting factor, while soil-derived inhibitors had minimal impact on PCR efficiency. For field applications, we conservatively set the pLoD at 1% contamination. Samples containing 0.5% contamination were classified as negative because, although occasional late amplification was observed (Cq 34.26 ± 0.29), these signals were not consistently reproducible across technical replicates and therefore did not meet our criteria for a true positive. No amplification was detected at 0.05% contamination.

## 4. Discussion

This study presents a validated, integrated molecular diagnostic framework to detect and quantify *C. beticola*. The framework addresses the urgent need for rapid, sensitive, field-deployable assays, especially as CLS management is complicated by reduced availability of effective plant protection products and widespread fungicide resistance. By combining portable real-time PCR (qPCR) platforms with digital droplet PCR (ddPCR), our dual-tiered strategy overcomes key limitations, including dependence on laboratory facilities, limited analytical sensitivity, and inadequate field applicability.

Portable qPCR assays, optimized for annealing at 62 °C and tested on two devices, achieved quantification efficiency (PCR efficiency 96–102%; R^2^ > 0.98) and detection limits (1 pg μL^−1^) comparable to laboratory-based systems and prior studies [10,11,36]. Notably, qPCR amplification exhibited pronounced sensitivity to minor temperature fluctuations, whereas absolute quantification by ddPCR remained statistically stable. This divergence highlights a potential practical advantage of ddPCR under the experimental conditions assessed here. ddPCR quantification relies on the partitioning of reactions into thousands of nanoliter-sized droplets and subsequent Poisson-based estimation of target concentration, rendering it less susceptible to variations in amplification kinetics compared to qPCR, where efficiency changes can directly alter Cq values and quantification accuracy [20,21]. However, ddPCR is not entirely immune to temperature variation; substantial deviations from optimal amplification conditions may still influence endpoint fluorescence intensities and target discrimination. Probit regression showed all platforms reached a 95% detection probability at the established LOD, meeting accepted validation criteria [37]. This confirms the diagnostic reliability and robustness of our integrated framework across diverse sample types. These findings add to the evidence that portable qPCR can deliver reliable quantitative results in the field, consistent with recent advances in plant pathology [17]. However, most portable qPCR research has focused on clinical or environmental samples [38,39], and practical on-site diagnostics for plant disease agents are still rare [38,39,40].

The ddPCR assay improves diagnostics by enabling absolute quantification at low target concentrations and showing resilience to matrix inhibitors [20,23]. As in prior studies [41,42], ddPCR provided superior accuracy and reproducibility at the lowest DNA levels, outperforming portable qPCR platforms in quantitative consistency when target abundance was minimal. This makes ddPCR valuable for applications requiring precise quantification near the detection limit. Comparative analysis showed near-perfect agreement between portable qPCR and ddPCR (all data within 95% limits), indicating both platforms are suitable for quantitative pathogen surveillance. This extends previous work [43] by confirming that portable qPCR is effective for rapid detection and quantification in field studies, addressing a critical need identified by Khan et al. [2] and Rangel et al. [3].

An important application of ddPCR is the early detection of *C. beticola* prior to the onset of visible symptoms. Because of its high sensitivity and quantitative precision, ddPCR can detect the pathogen when its abundance is still low, enabling identification of latent or pre-symptomatic infections earlier than conventional symptom-based diagnosis allows [22,23]. Early detection is crucial for effective disease surveillance and forecasting, as it allows timely management interventions before symptom development. When selecting between ddPCR and portable qPCR, practical diagnostic needs must be considered. Portable qPCR offers lower operational costs and simpler workflows, making it well suited for routine field surveillance. In contrast, ddPCR requires specialized instrumentation and consumables, resulting in higher per-sample costs and greater operational complexity. However, ddPCR excels in providing absolute target quantification with high precision, especially at low target concentrations and in complex sample matrices, without the need for external standard curves [20,23]. Therefore, ddPCR and portable qPCR should be viewed as complementary technologies. Portable qPCR is ideal for rapid, decentralized screening and routine surveillance, while ddPCR is particularly beneficial when high quantitative precision, low-level detection, or confirmatory analysis is required.

We rigorously validated specificity against a diverse panel of non-target fungal and bacterial isolates and across 20 *C. beticola* field isolates. The assay demonstrated robustness to natural sequence variation and did not produce cross-amplification. These results are consistent with Knight et al. [8] and further support the reliability of calmodulin as a molecular marker for *Cercospora* diagnostics, compared to less discriminatory loci.

A key innovation of this study is the systematic evaluation of two rapid DNA extraction protocols across multiple matrices, including fresh leaves, crop residues or soil, and seeds. Previous studies on *C. beticola* detection have primarily focused on the development and validation of specific molecular assays, including qPCR [8] and LAMP [9], using conventional laboratory-based DNA extraction procedures [7,10]. In contrast, the present study extends this diagnostic framework by evaluating rapid extraction procedures directly across diverse and potentially inhibitory matrices and by assessing their compatibility with both relative and absolute molecular quantification. The results demonstrated that the two extraction protocols produced comparable diagnostic outcomes, indicating that rapid extraction can provide DNA of sufficient quality for reliable *C. beticola* detection across a range of sample types. An additional contribution of this study is the integration of field-deployable DNA extraction with a dual-tiered molecular detection strategy, combining portable qPCR for rapid on-site relative quantification with droplet digital PCR (ddPCR) for precise absolute quantification. This combination extends previous *C. beticola* diagnostic approaches by providing not only sensitive detection but also a flexible framework for quantitative surveillance in complex environmental matrices. Protocol A, which uses the REDExtract-N-Amp Plant PCR Kit with a brief 95 °C incubation for 10 min, is particularly suitable for field deployment due to its simplicity and resistance to PCR inhibition, even in crude soil and seed extracts, as reported by Tomlinson et al. [11] and Opel et al. [44]. Protocol B, which employs a magnetic-bead-based kit (BN QuickPick Plant DNA Kit (Bio-Nobile, Finland)), achieves higher protein removal and similar qPCR detection limits but is more time-consuming and better suited for laboratory automation, consistent with the advantages described by Jie et al. [19]. Therefore, the choice of extraction method should be tailored to the specific diagnostic context, as also recommended by Schrader et al. [27]. Overall, this work advances existing *C. beticola* detection methods by linking rapid, matrix-compatible DNA extraction with complementary portable qPCR and ddPCR platforms, thereby providing a more versatile framework for field-to-laboratory surveillance. A pLoD was established in artificially inoculated soil at a 1% contamination threshold, which is directly relevant for field surveillance and management of overwintering inoculum. This finding aligns with epidemiological evidence that high inoculum densities are necessary for disease initiation [2]. It is comparable to detection thresholds reported for other soilborne and seedborne plant pathogens [33]. The data indicates that the framework is resilient to common PCR inhibitors in environmental samples, exhibiting minimal Cq shifts and no loss of diagnostic sensitivity, even with environmental inhibitors and sample variability, thereby confirming its suitability for field application.

Comparison with recent diagnostic innovations indicates that, although alternative methods such as LAMP, RPA, or CRISPR-based assays offer potential for rapid field detection, they frequently lack the quantitative rigor and multi-matrix validation demonstrated in this study [12,13,14,15,16]. Detection of *C. beticola* in both commercial and farmer-saved seed lots confirms the pathogen’s capacity for dissemination through multiple seed supply chains, highlighting the risk of disease spreading via diverse seed sources [4]. The portable qPCR-ddPCR framework presented here, which complies with MIQE and dMIQE guidelines, provides both high sensitivity and quantitative capability, distinguishing it from most published field-ready diagnostic tools.

Despite the robust performance demonstrated by this diagnostic framework, several methodological limitations must be acknowledged. First, although rapid extraction Protocol Aoffers a straightforward and effective method for field-based DNA preparation, the relatively low DNA purity achieved may restrict the long-term stability of DNA extracts and limit compatibility with downstream applications that require higher-quality DNA, such as genomic or sequencing-based analyses. Therefore, Protocol A is best regarded as an extraction strategy specifically optimized for rapid molecular detection, rather than as a universal DNA purification method. Second, as the assays detect pathogen DNA, they cannot differentiate between viable and non-viable *C. beticola* cells or propagules. As a result, DNA may persist following pathogen death, potentially leading to an overestimation of the infectious inoculum and associated disease risk in environmental samples [10]. Third, although specificity and performance were assessed using 20 field isolates, the consistency of detection across a broader spectrum of *C. beticola* genetic lineages from geographically diverse populations remains to be determined. Genetic variation in primer or probe binding regions could affect assay sensitivity and specificity; thus, broader validation using isolates representing diverse genetic backgrounds and geographic origins is warranted [4]. Finally, the current validation encompassed only a limited range of environmental conditions, which does not fully reflect the diversity of field settings in which the assay may be deployed. Variations in climatic zones, soil types, environmental inhibitors, and sample characteristics may influence extraction efficiency and assay reliability. Consequently, multi-location validation across contrasting environments will be essential to confirm the reproducibility and robustness of the diagnostic framework under a wider array of field conditions.

Implementation of this workflow enables actionable, in-field decision-making within hours, allowing crop protection specialists and field diagnosticians to respond rapidly to disease threats. This rapid turnaround is essential because CLS progresses quickly under favorable field conditions, and fungicide efficacy declines after symptom onset, supporting timely interventions and more effective disease management strategies.

## 5. Conclusions

This diagnostic framework represents a significant advancement for CLS management and plant disease diagnostics. It addresses critical scientific and practical gaps, demonstrates validated performance across diverse sample types and field conditions, and establishes a benchmark for rigorous, portable molecular diagnostics. Future research should focus on multiplexed detection of multiple pathogens, integration of fungicide resistance markers, and large-scale validation to improve scalability, predictive modelling, and sustainable crop protection strategies.

## Figures and Tables

**Table 1:** Statistical comparison of quantification cycle (Cq) values from portable qPCR and absolute concentrations (copies µL^−1^) measured by ddPCR among three different annealing temperatures. Data are presented as mean ± standard deviation (SD) of technical triplicates. Distinct letters indicate statistically significant differences according to Tukey’s HSD test (*p* < 0.05).

Annealing Temperature (°C)	qPCR ^a^			ddPCR ^b^		
	Cq ± SD	*p*-Value (ANOVA) and Tukey’s HSD Test		Copies µL^−1^ ± SD	*p*-Value (ANOVA) and Tukey’s HSD Test	
60	19.98 ± 0.21	<0.001	B	5482 ± 933	0.570	A
62	19.00 ± 0.22		A	6134 ± 358		A
64	20.04 ± 0.09		B	5869 ± 752		A

^a^ Cq values were calculated using a fixed fluorescence threshold of 16.76 RFU on a Franklin^®^ Three9 thermocycler (Biomeme, Inc., Philadelphia, PA, USA); ^b^ Absolute concentration (copies µL^−1^) was determined using Poisson distribution analysis of positive and total partitions on a QX200 Droplet Digital PCR system (Bio-Rad Laboratories).

## Data Availability

All the data generated in this study are provided within the manuscript or Supplementary Information files.

## Supplementary Materials

The following supporting information can be downloaded at https://www.mdpi.com/article/10.3390/jof12090686/s1, Figure S1: Optimization of annealing and extension temperatures for *C. beticola* detection. (A) qPCR amplification plots at 60, 62, and 64 °C with 1 ng·µL^−1^ gDNA input; negative (healthy plant, NC) and no-template controls (NTC) showed no amplification. (B) ddPCR amplitude plots showing droplet separation at each temperature; no fluorescence in controls. All experiments were performed in triplicate; Figure S2: Diagnostic validation of the qPCR assay across *C. beticola* isolates. Amplification plots were generated on qPCR platform 1 with 1 ng µL^−1^ DNA from 20 isolates collected from seven fields in Apulia, Southern Italy. All isolates were detected using a fixed fluorescence threshold of 24.52 relative fluorescence units (RFU). No fluorescence signal was detected in the non-template control (NTC); Table S1: Primers/TaqMan probe set used for *C. beticola* detection [8] (Knight et al., 2020); Table S2: Comparative assessment of average DNA yield and purity across diverse sample matrices using the REDExtract-N-Amp Plant PCR Kit (Sigma-Aldrich; protocol A) and the BN QuickPick Plant DNA Kit (Bio-Nobile; protocol B). This table also evaluates downstream *C. beticola* detection performance through portable qPCR (Cq) and absolute quantification by ddPCR (copies µL^−1^). Data are presented as means ± standard error from three independent extractions per matrix; Table S3: Mean DNA yield and purity of *Beta vulgaris* subsp. *vulgaris* seed lots extracted using the REDExtract-N-Amp Plant PCR Kit, along with corresponding detection and absolute quantification of *C. beticola* contamination in different seed lots via portable qPCR (Cq) and ddPCR (copies µL^−1^). Values represent the mean ± standard error from three independent extractions per sample; Table S4: Quantitative evaluation of DNA yield and purity from artificially inoculated soil matrices across a defined *C. beticola* contamination gradient, extracted using protocol A. The table further assesses assay sensitivity and detection limits using portable qPCR amplification (Cq). Values are reported as means ± standard error from three independent technical extraction replicates per contamination level.

## Abbreviations

The following abbreviations are used in this manuscript:

ANOVA	Analysis of variance
CLS	Cercospora leaf spot
CRISPR	Clustered regularly interspaced short palindromic repeats
CV	Coefficient of variation
Cas	CRISPR-associated
Cq	Quantification cycle
DNA	Deoxyribonucleic acid
E	Amplification efficiency
FAM	Carboxyfluorescein
HPLC	High-performance liquid chromatography
HSD	Honestly significant difference
IPC	Internal positive control
LAMP	Loop-mediated isothermal amplification
LOD	Limit of detection
LoA	Limits of agreement
MIQE	Minimum Information for Publication of Quantitative Real-Time PCR Experiments
NA	Nutrient agar
NC	Negative control
NCBI	National Center for Biotechnology Information
NTC	No-template control
PCR	Polymerase chain reaction
PDA	Potato dextrose agar
PNRR	Piano Nazionale di Ripresa e Resilienza
RPA	Recombinase polymerase amplification
R^2^	Coefficient of determination
SNP	Single Nucleotide Polymorphism
dMIQE	Digital Minimum Information for Publication of Quantitative Digital PCR Experiments
dUTP	Deoxyuridine triphosphate
ddPCR	Digital droplet polymerase chain reaction
dsDNA	Double-stranded deoxyribonucleic acid
gDNA	Genomic deoxyribonucleic acid
pLoD	Practical limit of detection
qPCR	Quantitative polymerase chain reaction
ΔCq	Shift in quantification cycles

## References

[1] Vogel J., Kenter C., Holst C., Märländer B. (2018). New Generation of Resistant Sugar Beet Varieties for Advanced Integrated Management of Cercospora Leaf Spot in Central Europe. Front. Plant Sci..

[2] Khan J., Rio L.E.D., Nelson R., Rivera-Varas V., Secor G.A., Khan M.F.R. (2008). Survival, Dispersal, and Primary Infection Site for *Cercospora beticola* in Sugar Beet. Plant Dis..

[3] Rangel L.I., Spanner R.E., Ebert M.K., Pethybridge S.J., Stukenbrock E.H., De Jonge R., Secor G.A., Bolton M.D. (2020). *Cercospora beticola*: The Intoxicating Lifestyle of the Leaf Spot Pathogen of Sugar Beet. Mol. Plant Pathol..

[4] Knight N.L., Pethybridge S.J. (2020). An Improved PCR Assay for Species-Specific Detection and Quantification of *Cercospora beticola*. Can. J. Plant Pathol..

[5] Skaracis G.N., Pavli O.I., Biancardi E. (2010). Cercospora Leaf Spot Disease of Sugar Beet. Sugar Tech.

[6] Secor G.A., Rivera V.V., Khan M.F.R., Gudmestad N.C. (2010). Monitoring Fungicide Sensitivity of *Cercospora beticola* of Sugar Beet for Disease Management Decisions. Plant Dis..

[7] Groenewald M., Groenewald J.Z., Crous P.W. (2005). Distinct Species Exist Within the *Cercospora apii* Morphotype. Phytopathology.

[8] Knight N.L., Koenick L.B., Sharma S., Pethybridge S.J. (2020). Detection of *Cercospora beticola* and *Phoma betae* on Table Beet Seed Using Quantitative PCR. Phytopathology.

[9] Shrestha S., Neubauer J., Spanner R., Natwick M., Rios J., Metz N., Secor G.A., Bolton M.D. (2020). Rapid Detection of *Cercospora beticola* in Sugar Beet and Mutations Associated with Fungicide Resistance Using LAMP or Probe-Based qPCR. Plant Dis..

[10] Schena L., Nigro F., Ippolito A., Gallitelli D. (2004). Real-Time Quantitative PCR: A New Technology to Detect and Study Phytopathogenic and Antagonistic Fungi. Eur. J. Plant Pathol..

[11] Tomlinson J.A., Dickinson M.J., Boonham N. (2010). Rapid Detection of *Phytophthora Ramorum* and *P. Kernoviae* by Two-Minute DNA Extraction Followed by Isothermal Amplification and Amplicon Detection by Generic Lateral Flow Device. Phytopathology.

[12] Anbazhagan P., Parameswari B., Anitha K., Chaitra G.V., Bajaru B., Rajashree A., Mangrauthia S.K., Yousuf F., Chalam V.C., Singh G.P. (2024). Advances in Plant Pathogen Detection: Integrating Recombinase Polymerase Amplification with CRISPR/Cas Systems. 3 Biotech.

[13] Kumar S., Singh R.M., Gadhave K.R. (2026). CRISPR–Cas Systems for Plant Virus Management: Detection, Surveillance, and Host Resistance. Front. Plant Sci..

[14] Zhao W., Chi Y., Ye M., Wang T., Xu A., Qi R. (2021). Development and Application of Recombinase Polymerase Amplification Assay for Detection of *Bipolaris sorokiniana*. Crop Prot..

[15] Ece E., Hacıosmanoğlu N., Güngen M.A., Tatlıses M.B., Eş İ., Hasançebi S., Inci F. (2025). In-Field Detection of Plant Pathogens Using Three-Dimensional-Printed Microneedles and a Portable Platform. ACS Sens..

[16] Rovetto E.I., Garbelotto M., Moricca S., Amato M., La Spada F., Cacciola S.O. (2024). A Portable Fluorescence-Based Recombinase Polymerase Amplification Assay for the Detection of Mal Secco Disease by *Plenodomus tracheiphilus*. Crop Prot..

[17] Knight N.L., Vaghefi N., Kikkert J.R., Bolton M.D., Secor G.A., Rivera V.V., Hanson L.E., Nelson S.C., Pethybridge S.J. (2019). Genetic Diversity and Structure in Regional *Cercospora beticola* Populations from *Beta vulgaris* subsp. *vulgaris* Suggest Two Clusters of Separate Origin. Phytopathology.

[18] Vaghefi N., Kikkert J.R., Bolton M.D., Hanson L.E., Secor G.A., Nelson S.C., Pethybridge S.J. (2017). Global Genotype Flow in *Cercospora beticola* Populations Confirmed through Genotyping-by-Sequencing. PLoS ONE.

[19] Jie J., Hu S., Liu W., Wei Q., Huang Y., Yuan X., Ren L., Tan M., Yu Y. (2020). Portable and Battery-Powered PCR Device for DNA Amplification and Fluorescence Detection. Sensors.

[20] Pinheiro L.B., Coleman V.A., Hindson C.M., Herrmann J., Hindson B.J., Bhat S., Emslie K.R. (2012). Evaluation of a Droplet Digital Polymerase Chain Reaction Format for DNA Copy Number Quantification. Anal. Chem..

[21] Yang R., Paparini A., Monis P., Ryan U. (2014). Comparison of Next-Generation Droplet Digital PCR (ddPCR) with Quantitative PCR (qPCR) for Enumeration of *Cryptosporidium oocysts* in Faecal Samples. Int. J. Parasitol..

[22] Raguseo C., Gerin D., Pollastro S., Rotolo C., Rotondo P.R., Faretra F., De Miccolis Angelini R.M. (2021). A Duplex-Droplet Digital PCR Assay for Simultaneous Quantitative Detection of *Monilinia fructicola* and *Monilinia laxa* on Stone Fruits. Front. Microbiol..

[23] Liu Y., Li J., Guo Z., Feng C., Gao Y., Liu D., Wang D. (2025). Development and Validation of a Droplet Digital PCR Assay for Sensitive Detection and Quantification of *Phytophthora nicotianae*. Front. Plant Sci..

[24] De Miccolis Angelini R.M., Habib W., Rotolo C., Pollastro S., Faretra F. (2010). Selection, Characterization and Genetic Analysis of Laboratory Mutants of *Botryotinia fuckeliana* (*Botrytis cinerea*) Resistant to the Fungicide Boscalid. Eur. J. Plant Pathol..

[25] Rotolo C., De Miccolis Angelini R.M., Pollastro S., Faretra F. (2016). A TaqMan-Based qPCR Assay for Quantitative Detection of the Biocontrol Agents *Bacillus subtilis* Strain QST713 and *Bacillus amyloliquefaciens* subsp. *plantarum* Strain D747. BioControl.

[26] Ophel-Keller K., McKay A., Hartley D., Herdina J., Curran J. (2008). Development of a Routine DNA-Based Testing Service for Soilborne Diseases in Australia. Austral. Plant Pathol..

[27] Schrader C., Schielke A., Ellerbroek L., Johne R. (2012). PCR Inhibitors—Occurrence, Properties and Removal. J. Appl. Microbiol..

[28] Ye J., Coulouris G., Zaretskaya I., Cutcutache I., Rozen S., Madden T.L. (2012). Primer-BLAST: A Tool to Design Target-Specific Primers for Polymerase Chain Reaction. BMC Bioinform..

[29] Bustin S.A., Benes V., Garson J.A., Hellemans J., Huggett J., Kubista M., Mueller R., Nolan T., Pfaffl M.W., Shipley G.L. (2009). The MIQE Guidelines: Minimum Information for Publication of Quantitative Real-Time PCR Experiments. Clin. Chem..

[30] Huggett J.F., Foy C.A., Benes V., Emslie K., Garson J.A., Haynes R., Hellemans J., Kubista M., Mueller R.D., Nolan T. (2013). The Digital MIQE Guidelines: Minimum Information for Publication of Quantitative Digital PCR Experiments. Clin. Chem..

[31] Huggett J.F., The dMIQE Group (2020). The Digital MIQE Guidelines Update: Minimum Information for Publication of Quantitative Digital PCR Experiments for 2020. Clin. Chem..

[32] Broeders S., Huber I., Grohmann L., Berben G., Taverniers I., Mazzara M., Roosens N., Morisset D. (2014). Guidelines for Validation of Qualitative Real-Time PCR Methods. Trends Food Sci. Technol..

[33] Xing M., Guan G., Zhang X., Sun H., Wang Z., Pang W., Piao Z., Yang X., Feng J., Liang Y. (2021). Spatiotemporal Quantification of *Plasmodiophora brassicae* Inoculum in Relation to Clubroot Development Under Inoculated and Naturally Infested Field Conditions. Plant Dis..

[34] Martin Bland J., Altman D.G. (1986). Statistical methods for assessing agreement between two methods of clinical measurement. Lancet.

[35] Armbruster D.A., Pry T. (2008). Limit of Blank, Limit of Detection and Limit of Quantitation. Clin. Biochem. Rev..

[36] Atallah Z.K., Bae J., Jansky S.H., Rouse D.I., Stevenson W.R. (2007). Multiplex Real-Time Quantitative PCR to Detect and Quantify *Verticillium dahliae* Colonization in Potato Lines That Differ in Response to *Verticillium* Wilt. Phytopathology.

[37] Forootan A., Sjöback R., Björkman J., Sjögreen B., Linz L., Kubista M. (2017). Methods to Determine Limit of Detection and Limit of Quantification in Quantitative Real-Time PCR (qPCR). Biomol. Detect. Quantif..

[38] Nguyen P.L., Sudheesh P.S., Thomas A.C., Sinnesael M., Haman K., Cain K.D. (2018). Rapid Detection and Monitoring of *Flavobacterium psychrophilum* in Water by Using a Handheld, Field-Portable Quantitative PCR System. J. Aquat. Anim. Health.

[39] Fernandez-Baca M.V., Castellanos-Gonzalez A., Ore R.A., Alccacontor-Munoz J.L., Hoban C., Castro C.A., Tanabe M.B., Morales M.L., Ortiz P., White A.C. (2024). A PCR Test Using the Mini-PCR Platform and Simplified Product Detection Methods Is Highly Sensitive and Specific to Detect *Fasciola hepatica* DNA Mixed in Human Stool, Snail Tissue, and Water DNA Specimens. Pathogens.

[40] Capron A., Stewart D., Hrywkiw K., Allen K., Feau N., Bilodeau G., Tanguay P., Cusson M., Hamelin R.C. (2020). In Situ Processing and Efficient Environmental Detection (iSPEED) of Tree Pests and Pathogens Using Point-of-Use Real-Time PCR. PLoS ONE.

[41] Hindson C.M., Chevillet J.R., Briggs H.A., Gallichotte E.N., Ruf I.K., Hindson B.J., Vessella R.L., Tewari M. (2013). Absolute Quantification by Droplet Digital PCR versus Analog Real-Time PCR. Nat. Methods.

[42] Whale A.S., Cowen S., Foy C.A., Huggett J.F. (2013). Methods for Applying Accurate Digital PCR Analysis on Low Copy DNA Samples. PLoS ONE.

[43] Strayer A.L., Jeyaprakash A., Minsavage G.V., Timilsina S., Vallad G.E., Jones J.B., Paret M.L. (2016). A Multiplex Real-Time PCR Assay Differentiates Four *Xanthomonas* Species Associated with Bacterial Spot of Tomato. Plant Dis..

[44] Opel K.L., Chung D., McCord B.R. (2010). A Study of PCR Inhibition Mechanisms Using Real Time PCR. J. Forensic Sci..

