# Supplementary material for: Portable qPCR and ddPCR Diagnostics for Cercospora Leaf Spot: Integrating Field and Laboratory Detection of Cercospora beticola in Beet
Source: J Fungi (Basel). 2026 Sep 12;12(9):686. doi: 10.3390/jof12090686 (PMC13608462; doi:10.3390/jof12090686)
Supplement: Supplementary file 1 [file jof-12-00686-s001.zip › Tables S1-S4.pdf]

Table S1. Primers/TaqMan probe set used for *C. beticola* detection (Knight et al., 2020).

Primer/Probe	Sequences (5' – 3')
CbCAL-Fw	GGCAGAGCTAACGACAGCAA
CbCAL-Rev	TTGTTGTCGGCGTCGACT
CbCAL-Probe	FAM-ACCCTCTGCGAATGTACTGAACTAACCTCG-BHQ1

Table S2. Comparative assessment of average DNA yield and purity across diverse sample matrices using the REDExtract-N-Amp Plant PCR Kit (Sigma-Aldrich; Protocol A) and the BN QuickPick Plant DNA Kit (Bio-Nobile; Protocol B). This table also evaluates downstream *C. beticola* detection performance through portable qPCR (Cq) and absolute quantification by ddPCR (copies μL^{-1}). Data are presented as means ± standard error from three independent extractions per matrix.

**Rapid extraction protocols**	**Samples**	**Spectrophotometric nucleic acid assessment**[a]			**Fluorometric dsDNA assessment**[b]	**qPCR**[c]	**ddPCR**[d]
		**ng µL^{-1}**	**λ260/λ280**[e]	**λ260/λ230**	**ng µL^{-1}**	**Cq**	**copies µL^{-1}**
**Protocol A**	Fresh leaves	132.9 ± 11.4	0.70 ± 0.04	0.74 ± 0.09	1.52 ± 0.18	32.89 ± 0.35	10.5 ± 0.8
	Crop residues with soil	36.4 ± 4.2	0.71 ± 0.05	1.45 ± 0.12	2.03 ± 0.22	29.36 ± 0.21	37.2 ± 2.1
	Seeds	24.4 ± 3.1	0.74 ± 0.03	0.69 ± 0.06	2.08 ± 0.25	38.72 ± 0.54	7.80 ± 0.9
**Protocol B**	Fresh leaves	9.5 ± 1.2	1.67 ± 0.09	0.91 ± 0.08	5.98 ± 0.45	31.16 ± 0.28	9.98 ± 1.1
	Crop residues with soil	15.4 ± 2.1	1.89 ± 0.14	0.82 ± 0.07	6.44 ± 0.52	31.32 ± 0.19	12.0 ± 1.4
	Seeds	18.1 ± 1.7	1.47 ± 0.08	0.78 ± 0.05	2.85 ± 0.31	35.02 ± 0.42	4.67 ± 0.6

[a]Total nucleic acid concentration and purity ratios were measured with a NanoDrop 2000 spectrophotometer; [b]double-stranded DNA (dsDNA) was quantified using a Qubit 2.0 fluorometer; [c] Cq values were calculated using a fixed fluorescence threshold of 50.99 RFU on a Biomeme Franklin® Three9 thermocycler; [d] Absolute concentration (copies μL^{-1}) was determined using a Bio-Rad QX200 Droplet Digital PCR system; [e] Although the low λ260/λ280 ratios observed indicate substantial protein carryover in Protocol A samples, the downstream PCR performance is not compromised. This is due to the PCR master mix's inherent tolerance for residual protein contaminants, ensuring reliable amplification; [e] Although the low λ260/λ280 ratios observed indicate substantial protein carryover in Protocol A samples, the downstream PCR performance is not compromised. This is due to the PCR master mix's inherent tolerance for residual protein contaminants, ensuring reliable amplification.

Table S3. Mean DNA yield and purity of *Beta vulgaris* subsp. *vulgaris* seed lots extracted using the REDExtract-N-Amp Plant PCR Kit, along with corresponding detection and absolute quantification of *C. beticola* contamination in different seed lots via portable qPCR (Cq) and ddPCR (copies µL^{-1}). Values represent the mean ± standard error from three independent extractions per sample.

Sample ID	Cultivar/variety description	Spectrophotometric nucleic acid assessment[a]			Fluorometric dsDNA assessment[b]	qPCR[c]	ddPCR[d]
		ng µL^{-1}	λ260/λ280[e]	λ260/λ230	ng µL^{-1}	Cq	copies µL^{-1}
**1**	Smooth green cutting leaf beet	721.3 ± 49.0	0.89 ± 0.02	0.14 ± 0.01	10.30 ± 0.30	ND[f]	ND
**2**	Green Swiss chard with white rib	711.4 ± 163.9	0.90 ± 0.07	0.15 ± 0.03	13.22 ± 3.48	ND	ND
**3**	Barese ribbed Swiss chard[g]	727.4 ± 98.0	0.89 ± 0.03	0.15 ± 0.02	17.70 ± 1.40	29.39 ± 0.87	22.70 ± 1.52
**4**	Leaf beet (Prius F1)	577.7 ± 99.1	0.86 ± 0.05	0.13 ± 0.02	11.83 ± 3.07	ND	ND
**5**	Barese ribbed Swiss chard	560.1 ± 21.9	0.83 ± 0.01	0.12 ± 0.00	13.94 ± 4.26	32.43 ± 1.36	2.59 ± 0.43
**6**	Barese ribbed Swiss chard	635.1 ± 116.9	0.87 ± 0.00	0.14 ± 0.01	12.05 ± 0.95	ND	ND
**7**	Barese ribbed Swiss chard[g]	521.4 ± 29.9	0.81 ± 0.02	0.12 ± 0.01	11.10 ± 0.80	30.37 ± 1.06	14.2 ± 1.21
**8**	Egnathia ribbed Swiss chard	668.7 ± 112.9	0.90 ± 0.04	0.14 ± 0.02	11.96 ± 2.14	ND	ND
**9**	Tonda di Chioggia leaf beet	654.4 ± 45.8	0.85 ± 0.00	0.14 ± 0.01	10.05 ± 0.25	ND	ND
**10**	Tonda di Chioggia ribbed Swiss chard	959.6 ± 13.9	1.07 ± 0.12	0.20 ± 0.04	10.55 ± 0.35	32.03 ± 1.14	3.20 ± 0.93
**11**	Smooth green cutting leaf beet	624.3 ± 5.7	0.88 ± 0.01	0.13 ± 0.00	11.20 ± 0.50	ND	ND
**12**	Green Swiss chard with broad silver rib	628.9 ± 150.2	0.86 ± 0.09	0.13 ± 0.02	13.16 ± 4.25	ND	ND
**13**	Barese ribbed Swiss chard	598.9 ± 171.6	0.84 ± 0.09	0.14 ± 0.04	13.96 ± 5.35	ND	ND

[a] Total nucleic acid concentration and purity ratios were measured using a NanoDrop 2000 spectrophotometer; [b] Double-stranded DNA (dsDNA) was quantified with a Qubit 2.0 fluorometer; [c] Cq values were calculated using a fixed fluorescence threshold of 46.58 RFU on a Biomeme Franklin® Three9 thermocycler; [d] Absolute concentration (copies µL^{-1}) was determined using a Bio-Rad QX200 Droplet Digital PCR system; [e] Although the low λ260/λ280 ratios observed indicate substantial protein carryover in Protocol A samples, the downstream PCR performance is not compromised. This is due to the PCR master mix's inherent tolerance for residual protein contaminants, ensuring reliable amplification; [f] ND: no amplification; [g] Farmer-saved seed lots obtained in 2024 (sample 3) and 2025 (sample 7); all others were commercial.

Table S4. Quantitative evaluation of DNA yield and purity from artificially inoculated soil matrices across a defined *C. beticola* contamination gradient, extracted using protocol A. The table further assesses assay sensitivity and detection limits using portable qPCR amplification (Cq). Values are reported as means ± standard error from three independent technical extraction replicates per contamination level.

Contamination level (%)	Spectrophotometric nucleic acid assessment[a]			Fluorometric dsDNA assessment[b]	qPCR[c]
	ng µL^{-1}	λ260/λ280	λ260/λ230	ng µL^{-1}	Cq
**100**	1001.66 ± 31.91	0.91 ± 0.01	0.20 ± 0.00	22.33 ± 0.84	26.32 ± 0.31
**75**	794.88 ± 32.76	0.90 ± 0.01	0.19 ± 0.00	17.27 ± 1.13	27.05 ± 0.12
**50**	678.73 ± 70.99	0.89 ± 0.00	0.18 ± 0.01	16.77 ± 1.12	27.29 ± 0.13
**25**	473.90 ± 25.02	0.84 ± 0.01	0.14 ± 0.00	13.60 ± 0.32	28.75 ± 0.29
**10**	362.23 ± 7.54	0.81 ± 0.01	0.23 ± 0.10	11.70 ± 0.68	29.49 ± 0.33
**5**	300.42 ± 5.80	0.77 ± 0.01	0.33 ± 0.01	9.33 ± 0.59	31.71 ± 0.15
**1**	351.09 ± 10.19	0.70 ± 0.01	0.53 ± 0.03	6.00 ± 0.24	33.07 ± 0.29
**0.5**	346.19 ± 9.06	0.71 ± 0.01	0.49 ± 0.03	6.07 ± 0.30	34.26 ± 0.29 (NA[d])
**0.1**	342.22 ± 3.71	0.71 ± 0.00	0.48 ± 0.02	5.65 ± 0.23	36.98 ± 0.11 (NA)
**0.05**	331.62 ± 5.78	0.71 ± 0.00	0.45 ± 0.03	5.66 ± 0.42	ND[e]

[a] Total nucleic acid concentration and purity ratios were measured using a NanoDrop 2000 spectrophotometer; [b] Double-stranded DNA (dsDNA) was quantified with a Qubit 2.0 fluorometer; [c] Cq values were calculated using a fixed fluorescence threshold of 18.91 RFU on a Biomeme Franklin® Three9 thermocycler; [d] NA: no amplification signal meeting the predefined assay detection criteria; [e] ND: complete absence of amplification.
